# Acceptability and appropriateness of a perinatal depression preventive group intervention: a qualitative analysis

**DOI:** 10.1186/s12913-020-5031-z

**Published:** 2020-03-07

**Authors:** Alicia Diebold, Melissa Segovia, Jessica K. Johnson, Aria Degillio, Dana Zakieh, Hee Jin Park, Kenneth Lim, S. Darius Tandon

**Affiliations:** 1 Center for Community Health, Institute for Public Health and Medicine, Northwestern University Feinberg School of Medicine, 750 N Lake Shore Drive, Suite 643, Chicago, IL 60611 USA; 2 Center for Community Health, Institute for Public Health and Medicine, Northwestern University Feinberg School of Medicine, 750 N Lake Shore Drive, Suite 680, Chicago, IL 60611 USA

**Keywords:** Perinatal depression, Home visiting, Implementation, Qualitative analysis

## Abstract

**Background:**

Perinatal depression is a prevalent public health concern. Although preventive interventions exist, there is limited literature on the acceptability and appropriateness of these interventions, especially those delivered by paraprofessionals. The Mothers and Babies Program (MB) is a group-based perinatal depression preventive intervention delivered prenatally. A cluster-randomized controlled trial examined the acceptability, appropriateness, and effectiveness of MB delivered by mental health professionals compared to paraprofessional staff from home visiting programs.

**Methods:**

The full study enrolled 874 pregnant women. Fifty-three facilitators were trained and delivered the MB intervention to women in one of seven states in the United States. Semi-structured interviews were attempted with a randomly-selected subset of the full sample of pregnant women who received the MB intervention and with all facilitators. Specifically, interviews were conducted with 88 women who received the MB group intervention (45 in the paraprofessional-led arm and 43 in the mental health professional-led arm) and 46 women who facilitated the groups (27 home visiting staff and 19 mental health professionals). Interviews were conducted over the phone in English or Spanish and audio recorded. The recordings were translated into English, as needed, and transcribed. Thematic analysis was conducted using NVIVO to identify key themes related to intervention acceptability and appropriateness. Similarities and differences between study arms were explored.

**Results:**

Clients and facilitators found the MB content and group format acceptable. Challenges included maintaining group attendance, transportation issues, and managing group discussion. Overall, facilitators found the intervention appropriate for pregnant clients with some challenges presented for clients in crisis situations, experiencing housing instability, and with literacy and learning challenges. Participants provided suggestions for improvement, both for the course content and implementation. There were no significant differences found between study arms.

**Conclusions:**

Overall, clients and facilitators enjoyed MB irrespective of study arm, and facilitators found the intervention appropriate for the population. These findings add to the qualitative literature on perinatal depression preventive interventions, specifically those delivered by paraprofessionals.

**Trial registration:**

This trial is registered on ClinicalTrials.gov (Initial post: December 1, 2016; identifier: NCT02979444).

## Contributions to the literature

- Paraprofessionals are being used more frequently to fill gaps in maternal and child health services, especially in underserved populations; however, little is known about the acceptability and appropriateness of paraprofessionals in delivering interventions, specifically postpartum depression preventive interventions from the perspective of clients and interventionists.
- Our findings show that clients found a perinatal depression preventive intervention, the Mothers and Babies Program, acceptable regardless of whether it was implemented by a paraprofessional or mental health professional; and interventionists found it appropriate for the population with a few exceptions.
- Paraprofessionals may be a viable and cost-effective option for delivering maternal and child health interventions in under-resourced areas.
- The Mothers and Babies Program is acceptable and appropriate for clients in a home visiting setting.

## Background

Perinatal depression is prevalent in the United States, affecting as many as one in seven women during pregnancy and up to 1 year postpartum [1]. These rates are higher in vulnerable groups (e.g., low socioeconomic status, adolescents, low social support) of women [2]. Perinatal depression has well-established negative impacts for mothers and their children [3]. Recent years have seen a growing emphasis placed on screening perinatal women for depression, including recommendation statements from national professional organizations [4–6]. The identification of women needing perinatal mental health services requires the availability of interventions that can treat women already experiencing a depressive episode and prevent depression among women exhibiting risk factors for perinatal depression. Several preventive interventions have been developed and tested, with a handful showing effectiveness in preventing the onset and worsening of perinatal depression [7–9].

One intervention that has shown to be effective in preventing the worsening of depressive symptoms and onset of new depressive episodes is the Mothers and Babies Program (MB) [7]. MB is based on principles of cognitive behavior therapy (CBT) and attachment theory. Previous studies on the MB group modality led by mental health professionals (MHPs) established the effectiveness of the intervention in reducing depressive symptoms and preventing the onset of new depressive episodes [9–12]; however, the acceptability and appropriateness of MB for facilitators delivering the intervention and clients receiving the intervention has not been examined in-depth. This is consistent with a gap in the literature for similar perinatal depression preventive interventions. A systematic review [8] identified 29 articles from 22 unique studies that pertained to clients’ and providers’ perceptions and attitudes of postpartum depression preventive interventions [13]. However, the authors noted that a limitation of this review was the majority of the included studies were of moderate to low quality due to methodological limitations (i.e., a lack of details related to data collection, inadequate data collection methods, lack of information regarding researcher reflexivity, or a lack of detailed and rich data) and/or limited coherence of the findings [14]. This points to a need for further research on client and provider perceptions on postpartum preventive interventions to aid in the generalization of results and development of future interventions.

There is also a need for more qualitative research aimed at understanding the acceptability and appropriateness of maternal and child health interventions involving lay health workers. In a systematic review looking at the effects of lay health workers on maternal and child health outcomes, 82 trials were identified. Of these 82 trials, nearly two-thirds had no qualitative research conducted alongside the trial, and only eight looked at the perceptions of both trial participants and the interventionists [15]. The results of this systematic review highlight the need for more qualitative data to be collected to aid in identifying reasons for heterogeneous findings related to intervention efficacy.

Data reported here comes from a larger cluster-randomized controlled trial (C-RCT) that tested the effectiveness of the MB group modality facilitated by lay health workers, referred to here as paraprofessionals, compared to MHPs. One aim of this study was to examine client and facilitator perceptions, specifically regarding acceptability and appropriateness, of MB when it is implemented in home visiting (HV) programs and delivered by paraprofessional HV staff and MHPs. This was done through the analysis of qualitative data collected through semi-structured interviews with both intervention clients and facilitators. The qualitative phase of the larger C-RCT is the largest qualitative analysis of MB, and strives to add to the literature on client and provider perceptions of perinatal depression preventive interventions, as well as the broader literature on the implementation of maternal and child health interventions by paraprofessionals.

In this manuscript, we explore the acceptability and appropriateness of the MB group modality for HV programs and clients using qualitative data through the lens of the taxonomy of implementation outcomes presented by Proctor et al. (2011) [16]. In this taxonomy, acceptability refers to the degree to which the intervention is “agreeable, palatable, or satisfactory,” and appropriateness is “perceived fit, relevance, or compatibility, of the…practice for a given practice setting, provider, or consumer.” [16] Specifically, this manuscript will report on: 1) client and facilitator perceptions regarding the acceptability and appropriateness of the MB content; 2) client and facilitator perceptions regarding the acceptability and appropriateness of the MB group format and implementation; and, 3) differences and similarities on MB acceptability and appropriateness between study arms (MB delivered by HV staff or by MHPs). In doing so, we have generated insights on the implementation of a perinatal depression preventive intervention delivered by individuals with varying professional training.

## Methods

### Study design

The larger C-RCT took place from September 2016 to September 2019. Participants were referred from 37 HV programs in seven states—Ohio, Michigan, Illinois, Missouri, Minnesota, Iowa, and West Virginia—with a total of 874 clients (405 HV staff-led arm, 310 MHP-led arm, and 159 Control) and 53 group facilitators (32 paraprofessional HV staff and 21 MHPs) enrolled. The C-RCT compared the effectiveness of the MB group intervention delivered by paraprofessional HV staff and MHPs. Home visiting programs were randomized into one of three study arms: (1) MB delivered by a paraprofessional HV staff member; (2) MB delivered by a MHP; or, (3) usual HV services without MB. Jensen et al. (2018) provides additional details of the study protocol.

### Mothers and Babies Program group intervention

The MB group intervention consists of six sessions comprised of three core CBT areas: thoughts, pleasant activities, and social support. For the purposes of this study, we referred to the delivery of the six-session MB intervention as a “cohort.” Intervention sessions for a cohort were conducted at the HV program office or another community location. Date and time of intervention sessions were specific to each cohort in an attempt to maximize attendance among group members. Attendance for group sessions was, on average, three women and the average length of a group session was 86 min. Instructor and participant manuals were used to deliver MB. Instructor manuals consisted of a script to guide the facilitator’s delivery of the intervention, as well as activities to prompt group discussion. Participant manuals were given to all clients at the first intervention session and contained worksheets that aligned with content in the instructor manual. Participant manuals included activities for participants to complete during a MB session, vignettes to depict certain concepts, and a “Quick Mood Scale” (QMS) for participants to track their moods at home (see Additional Files 1 & 2 for examples). As part of the research study, HV programs were provided financial support to offset costs related to participant transportation to attend group sessions, child care, and snacks. Financial assistance varied by site and was concordant with the needs of each program.

### Study participants

#### Mothers and Babies Program group intervention facilitators

All 53 facilitators who implemented at minimum one cohort of the intervention were approached to participate in a semi-structured interview, with 46 facilitators (27 HV staff and 19 MHPs) completing interviews. Two facilitators declined to complete an interview and five were unresponsive to outreach. Facilitators led anywhere from 1 to 12 cohorts, with the average leading 2.5 cohorts. Paraprofessionals were recruited from participating HV programs to be facilitators. Paraprofessionals had no advanced degrees in mental health or a related field. Mental health professionals were recruited from HV programs, or from state professional organizations for programs that did not have a staff member who met the qualifications. Qualifications to be a MHP were having a master’s degree in a related field (i.e., social work, early childhood development) and at least 5 years’ experience working with children and families. One exception to this was made for a facilitator who did not have a master’s degree, but was actively working toward one and had nearly 10 years of experience. All facilitators attended a day or day and a half MB training conducted by the principal investigator (PI) and participated in weekly supervision phone calls with the PI while facilitating their first cohort. All facilitators consented to take part in the study via an online consent form. Table 1 shows demographic information of interviewed facilitators.

**Table 1:** Demographics of Facilitators

	Paraprofessional Home Visitors (*n* = 27)	Mental Health Professionals (*n* = 19)	Total (*N* = 46)
**Race: n (%)**			
Black/African American	9, 33.3%	3, 15.8%	12, 26.1%
White/Caucasian	11, 40.7%	11, 57.9%	22, 47.8%
Hispanic/Latino	6, 22.2%	5, 26.3%	11, 23.9%
Asian American	0, 0%	0, 0%	0, 0%
Native American	0, 0%	0, 0%	0, 0%
Bi-racial	0, 0%	0, 0%	0, 0%
Missing/Unknown	1, 3.7%	0, 0%	1, 2.2%
**Education (Highest Achieved): n (%)**			
High School	1, 3.7%	0, 0%	1, 2.2%
Associate Degree	3, 11.1%	0, 0%	3, 6.5%
Bachelor’s Degree	18, 66.7%	1, 5.3%	19, 41.3%
Master’s Degree	5, 18.5%	16, 84.1%	21, 45.6%
Professional Degree	0, 0%	1, 5.3%	1, 2.2%
Doctoral Degree	0, 0%	1, 5.3%	1, 2.2%
**Years Practicing in Field: n (%)**			
1–2	10, 37%	2, 10.5%	12, 26.1%
3–5	8, 29.6%	3, 15.8%	11, 23.9%
6–10	4, 14.8%	5, 26.3%	9, 19.6%
11–15	3, 11.1%	4, 21.1%	7, 15.2%
> 15	2, 7.4%	4, 21.1%	6, 13.0%
Missing/Unknown	0, 0%	1, 5.3%	1, 2.2%

#### Mothers and Babies Program group intervention clients

Eligibility criteria to participate in the larger C-RCT included being ≥16 years old, being ≤33 weeks gestation upon referral, and speaking either English or Spanish. Clients were referred from participating HV programs and the surrounding communities. Referred clients were screened for eligibility, and if eligibility criteria was met, research staff would describe the study and ascertain interest in participation. If a client agreed to participate in the research study, informed consent was obtained either over the phone or through an online consent form. In total, 874 clients were enrolled in the study, with 715 of those enrolled into one of the two MB group intervention arms. Eighty-eight intervention clients were randomly selected from the intervention arms (45 who received MB delivered by HV staff and 43 who received MB delivered by a MHP) to participate in an interview. The sample was large enough for themes to emerge, but small enough that it was manageable given staff time and resources. Given the aim of the interviews was specifically related to the intervention, study control participants were not interviewed. Table 2 shows demographic information of interviewed clients.

**Table 2:** Demographics of Clients

	**Study Arm**		Total (*N* = 88)
	Paraprofessional Home Visitor Facilitated Groups (*n* = 45)	Mental Health Professional Facilitated Groups (*n* = 43)	
**Race: n (%)**			
Black/African American	22, 48.9%	14, 32.6%	36, 40.9%
White/Caucasian	8, 17.8%	11, 25.6%	19, 21.6%
Hispanic/Latino	11, 24.4%	13, 30.2%	24, 27.3%
Asian American	1, 2.2%	1, 2.3%	2, 2.3%
Native American	3, 6.7%	1, 2.3%	4, 4.5%
Bi-racial	0, 0%	3, 7.0%	3, 3.4%
**Education (Highest Achieved): n (%)**			
1st-8th Grade	2, 4.4%	1, 2.3%	3, 3.4%
9th–12th Grade, No Diploma	4, 8.9%	13, 30.2%	17, 19.3%
High School Diploma or GED	16, 35.6%	11, 25.6%	27, 30.7%
Some College	12, 26.7%	10, 23.3%	22, 25.0%
College Degree	11, 24.4%	7, 16.3%	18, 20.5%
Missing/Unknown	0, 0%	1, 2.3%	1, 1.1%
**Household Income: n (%)**			
< $24,999	35, 77.8%	24, 55.8%	59, 67.0%
$25,000–$49,999	8, 17.8%	14, 32.6%	22, 25.0%
$50,000–$74,999	2, 4.4%	1, 2.3%	3, 3.4%
$75,000–$99,999	0, 0%	0, 0%	0, 0%%
> $100,000	0, 0%	2, 4.7%	2, 2.3%
Missing/Unknown	0, 0%	2, 4.7%	2, 2.3%
**First-time Mother: n (%)**			
Yes	17, 37.8%	14, 32.6%	31, 35.2%
No	28, 62.2%	28, 65.1%	56, 63.6%
Missing/Unknown	0, 0%	1, 2.3%	1, 1.1%
**Age: Mean**			
	27.4	26.7	27.0

### Researcher characteristics and reflexivity

The PI of the C-RCT is an expert in the field of perinatal depression, leading several studies related to the implementation of MB. Staff involved in the qualitative analysis included a project manager, two research coordinators, four research assistants, and one master’s-level intern. All staff have backgrounds in a related field such as early childhood development, public health, social work, or psychology. Client interviews were conducted by one of four research staff members—two of whom were bilingual. The staff had varying levels of previous contact with intervention clients during the screening and enrollment process of the study. Facilitator interviews were conducted by the study’s Implementation Coordinator, who had previous communication with the facilitators during the implementation phase of the intervention. These existing relationships may have impacted both the participants’ level of engagement in an interview and openness in their responses.

### Data collection

Semi-structured interviews were conducted over the phone by one of the research staff members mentioned above. Separate semi-structured interview guides were created for the client and facilitator interviews asking about their experiences with MB (see Additional Files 3 & 4). Facilitator interviews were conducted in English. Client interviews were conducted in the participant’s preferred language (English or Spanish). All interviews were audio-recorded, uploaded to a secure shared drive, and professionally transcribed. Interviews conducted in Spanish were translated into English during the transcription process. Consent to be contacted for and participate in an interview was obtained during the initial consenting process for the study for both facilitators and clients. All materials used in the study were approved by the Institutional Review Board at Northwestern University. Each client and facilitator was given an identification number used for saving and storing interview recordings and transcripts. The key for these identification numbers was housed in a secure web application, Research Electronic Data Capture (REDCap) [17].

Clients were contacted for an interview within a week of completing the intervention and interviews were scheduled within the next month to ensure a more accurate recollection of their experience with the intervention. If a client could not be reached within that timeframe, another client was selected to participate in an interview. Clients received a $20 gift card for participating in an interview. Facilitator interviews were scheduled, on average, within 3 months after the completion of the implementation of groups. Client and facilitator interviews were conducted between April 2017 and October 2018.

In addition to the interviews, clients were also asked to complete a Participant Feedback Form at the end of every group session. The forms asked clients to rate (using a three-point scale) how much they enjoyed that day’s session, how well they understood the information presented in the session, and how often they thought they would use the skills and information presented in the session.

### Analysis

Prior to analysis, the research team developed one codebook for the facilitator interviews and one for client interviews with codes and sub-codes related to the interview questions and key areas of interest for the researchers. The codebooks were regularly reviewed and updated throughout the coding process as new codes and sub-codes emerged across transcripts (see Additional Files 5 & 6 for the final codebooks). Four members of the research team (AD, KL, MS, HP) coded interview transcripts. Prior to individual coding, 10% of the transcripts were coded by all four team members and reviewed to ensure consistency and reliability among coders. Any discrepancies were discussed and resulted in a consensus among all coders.

Coding and analysis were done using NVIVO Pro (version 11). A thematic analysis approach was used in this study [18]. This approach was chosen for its flexibility, ease of use, and ability to work with a large data set. Once coding was completed, data was cleaned and organized into framework matrices. Analysis was conducted by four staff members (MS, HP, AD, JJ) and took place from July 2018 to April 2019. MS and HJ separately analyzed the framework matrices to identify fundamental themes and AD and JJ reviewed results to ensure validity.

## Results

The results presented here focus on the most common themes related to the acceptability and appropriateness of the MB content and group format (see Table 3). Although other themes emerged from the dataset, this manuscript focuses on the specific study aim related to client and facilitator perceptions of the acceptability and appropriateness of the MB intervention when delivered by paraprofessional HV staff and MHPs.

**Table 3:** Frequency of Themes by Facilitators and Clients

**Theme**	**Sub-Themes**	**Facilitator Frequency: n (%)** ***N* = 46**	**Client Frequency: n (%)** ***N* = 88**
**Clients and facilitators found the Mothers and Babies content acceptable and appropriate.**	Facilitators played a valuable role in relaying the information.	3(6.5%)	22(25.0%)
	Clients found the tools and vignettes especially useful.	20(43.5%)	17(19.3%)
**There were barriers and motivators to attendance.**	Barriers to attendance included challenges experienced during pregnancy, family crises, scheduling conflicts, and transportation.	13(28.3%)	32(36.4%)
	Assistance and access to transportation and child care played a key role in increasing client attendance.	4(8.7%)	13(14.8%)
**Clients found the group format acceptable.**	Clients found the group size just right.	N/A	44(50.0%)
	Being in a group of other pregnant women helped to normalize thoughts and feelings.	29(63.0%)	20(22.7%)
	There were feelings of sadness at the completion of group.	2(4.3%)	6(6.8%)
**Facilitators raised concerns with the appropriateness of the group format for certain populations.**	Facilitators found it challenging to engage women who were experiencing crises, housing instability, and/or trauma.	11(23.9%)	N/A
	Facilitators found it difficult to implement with clients who were illiterate, had literacy challenges, or learning disabilities.	6(13.0%)	
	Facilitators found it challenging to manage discussions during group.	21(45.7%)	

Facilitators’ overall feedback and experiences related to the group format were positive and reflected a strong acceptability of the MB materials. This was demonstrated in the rating given by facilitators on the overall program, including ease of implementation and course content. Facilitators’ mean rating of the MB program was a 4.44 rating on a scale of 1–5, where “1″ was “poor” and “5″ was “excellent.” Likewise, clients’ overall feedback of the group format and material was also positive. This was reflected in Participant Feedback Forms completed by clients at the end of each group session. Taking an average of the scores, 94.9% of clients stated they enjoyed that day’s session; 97.6% of clients reported they understood the information; and, 87.4% of clients marked that they thought they would use the skills and information presented in that day’s session “often.”

### Mothers and Babies content

#### Clients and facilitators found the Mothers and Babies content acceptable and appropriate

Overall, facilitators and clients described the content of MB as easy to understand, useful, relatable, and universal.

“Yeah, it wasn’t hard to read at all…there was other examples and there was activities that you can do as well, so it’s not like you’re just left there like, ‘I don’t know how to do this,’ or, ‘I really didn’t understand the activity for today or the session for today or the activity that you would have to do at home,’ and that was very helpful as well, actually paying attention to your days and how everything goes.” [Client]

Clients reported consistent understanding and satisfaction with the content of all three MB modules (pleasant activities, thoughts, and social support). The vast majority of the clients discussed using skills from one or more of the modules in their daily lives.

“I try to look back on the pleasant activities and then also feel like moods and outer reality, I try to keep it not so negative inside. I mean I don’t know when I first started out, I had like 13 negative thoughts. Then I got it down to like six or five I remember.” [Client].

Clients denoted the valuable role that their facilitator played in the acceptability of the content. Although the content was described as straightforward by clients and facilitators alike, clients repeatedly highlighted how their facilitator provided both meaning and context as needed during the group sessions*.*

“I think the reason why—I know me in particular—received the information so well is because the facilitator was very engaging. You can have facilitators who make the content dry, drab—you know, it feels like our facilitator actually kept it interesting…We read, just it didn’t feel like we were just going through the motions. There was always an opportunity for feedback and insight and things of that nature that made us able to apply the content to our own lives a little bit more. We always were able to place things into perspective.” [Client]

Clients brought up specific tools from the curriculum they found most helpful; specifically the QMS and vignettes used to introduce each CBT module (see Additional Files 1 & 2 for reference). Clients noted that the experience of tracking their moods daily helped them draw connections between their thoughts, activities, and/or social supports and their moods. A client described the impact the QMS had on her mood:

“...it helped me monitor my moods and my activities. I noticed when I wasn’t doing activities that my mood was a little low. If I kept busy, my mood was a little better.” [Client]

The vignettes were referred to repeatedly in a very favorable light by both clients and facilitators. The vignettes helped clients identify thought patterns and behaviors, and also helped clients draw connections between actions and their moods.

“There are examples on some of them [vignettes], where they showed an example of one person and the other person. One person was negative about their day and just didn’t want to do anything and the other one started off negative and then they try to get their day better by doing things. And well sometimes I feel like that. Sometimes I just don’t want to get up. It just takes me a while to figure out how to get myself to feel better.” [Client]

A significant portion of facilitators felt that the majority of MB content was appropriate, irrespective of client demographics, with some facilitators and clients noting MB was relevant to other family and community members as well as to their own personal lives.

“...So the fact that it can relate to anybody—it doesn’t matter what your race, gender, age, sexual orientation is—it pertains to everybody that’s going through this new chapter of their life. Whether it’s having their first child, or having a second or a third child, everybody goes through the change in life and I just thought it helped us come together. Just because we’re all different doesn’t mean that we don’t have some common similarities and Mothers and Babies I think really pulled out the similarities in the groups." [Facilitator]

### Group format and implementation

#### Attendance barriers and motivators

There were several challenges reported by clients and facilitators related to attendance. Some facilitators shared frustrations with cohorts or individual sessions that were unable to get started due to low attendance. Facilitators and clients noted that the very characteristics of the population that was recruited for groups created challenges in attendance. For example, challenges during pregnancy, such as fatigue and medical complications, impacted the women’s ability to attend groups.

“I just was not feeling well. Sometimes I would come to class and not feel my best, but I would still stick it out. But, it was kind of like a struggle to drag myself there only because I was not feeling well...” [Client]

Despite efforts to schedule cohorts at the most convenient times for clients, there were multiple factors that affected their availability to attend groups. The most prevalent factors that impacted attendance, mentioned by clients and facilitators, were family crises and scheduling conflicts with work and school. Clients also noted having to skip sessions to take care of family members or sick children.

“I also feel like that’s another deterrent for other people because of the time because if you’re working full-time and things like that, and you don’t have a flexible schedule, it’s kind of hard to get that two to two and a half hours once a week in the middle of the day.” [Client]

Additionally, challenges related to transportation to and from groups were identified, including access to reliable transportation, having to travel long distances, and having to travel during rush hour traffic in urban areas.

“You know, the challenges, transportation. You know, getting there. Because sometimes I do—I live far from the office [HV Program] but that part is challenging living far out from the office. And ya’ll have it…people come get us back and forth. That’s—I know it’s challenging for me and them because they gotta worry about gas and we gotta worry about our safety.” [Client]

Assistance and access to transportation and child care services played a key role in client attendance. Free child care, which was provided at the majority of the sites, was helpful even for first time mothers who were taking care of younger siblings or other young family members. All clients deemed this valuable, yet facilitators noted the increased time needed to coordinate this service and transition the children to child care, in order for groups to start on time.

“I thought the child care was perfect because sometimes we have two or three children that we can’t leave unsupervised or that we don’t have someone who can take care of them. I thought that was perfect.” [Client]

#### Clients found the group format acceptable

The group environment played a key role in encouraging women to engage in intervention activities. Clients noted that an intimate, welcoming, and confidential space impacted their decisions to stay and share during group sessions.

“At first, I was kind of skeptical because I am kind of a quieter person, so I was kind of skeptical about opening up and just revealing personal information about myself, but I knew that the group was confidential, and it was told to us that the group was confidential and nothing that we said was going to be used outside or anything, so that kind of made me open up." [Client]

The majority of the clients felt their cohort size fit in a “Goldilocks band” where the group had been large enough to learn from peers, yet small enough to share freely without feeling rushed. Some clients voiced a desire to have additional women in group, yet others voiced that they would not have felt as comfortable if there had been more women in the session. No clients expressed concern with the group size being too large.

"It was good at four because we could all share a little bit here and there. If it was more than six, then it would take too long because it’s six people trying to say something. And you don’t know how long you would have. And you don’t want to rush certain things." [Client]

Clients emphasized the important role that their peers played in the group experience. Having a space to meet other women who were pregnant and experiencing similar challenges was very meaningful. Additionally, meeting women outside of their home helped address feelings of isolation and loneliness. The experiences shared in group helped clients form friendships and valuable social support networks with peers. Clients and facilitators noted that the creation of social support amongst peers was one of the most significant outcomes of the groups.

“I think a lot of it was just being an intimate group of women...Even though we all come from completely different backgrounds, me being a first-time mom who works full-time and one having four kids and then the other one being a stay-at-home mom but yet we all shared a lot of the same day-to-day struggles." [Client]

“Even though we didn’t know each other you could—they showed care. And that was the biggest thing for me...It’s just nice to have—that you still have people out there that are concerned of your wellbeing...They would just say, ‘Happy you came. Good seeing you.’ That was another thing that kept me coming by, getting the little support from them, of keeping yourself encouraged to keep coming." [Client]

Numerous women noted that their main motivation in attending MB groups was to become “better mothers.” They emphasized the value of pregnancy and parenting experiences shared by peers during group. The conversations during sessions helped normalize thoughts and information for clients. Facilitators noted that the normalization of certain feelings also helped decrease feelings of isolation, assuring mothers that they are not alone. It was noted that the normalization of specific feelings was a byproduct of a group environment and would have been difficult to address in a one-on-one setting.

“And one of the moms, her eyes lit up, and she was like, ‘...I thought I was the only one, and I felt like I was a horrible mother because I wasn’t initially just ecstatic.’ So, then we went into those kinds of conversations, and just having them realize that they’re not the only ones experiencing whatever it is they’re experiencing, I think that was just wonderful for them.” [Facilitator]

Numerous clients voiced that the cohort went by “fast.” Various clients noted feeling sad with the completion of their cohort. The majority of clients voiced that the length of the sessions was ideal, with some mothers voicing that the sessions could have gone on a bit longer. Only one client noted that a cohort could have been shorter than six sessions.

"And I know that a lot of people were upset when the classes came to an end because we had all felt such a bond together—that we didn’t know what to do with our Wednesdays after the class ended." [Client]

#### Challenges related to the appropriateness of the Mothers and Babies Program group intervention and suggestions for improvement

Facilitators voiced challenges with the implementation of the group modality with clients who were experiencing crises, housing instability (including homelessness, couch surfing, and precarious living situations), and/or trauma. Several facilitators suggested the program incorporate examples that discussed these challenges into the examples and vignettes found in the manuals to help facilitators address them. A couple of facilitators also suggested incorporating more resources for facilitators to give to clients in the program.

“I found a lot of the moms were homeless, and I didn’t realize before this group...I didn’t realize that so many moms were homeless. For some reason, they opened up more in the Mothers and Babies group and told me that they were homeless, a lot of them living in shelters and pregnant, or living in a car, or they were squatters living in homes, and just to be able to address those issues...Well, I think each group facilitator should at least be aware that those stressors are real and that they should have a resource to refer to because it will come up. It came up in every group I had...So, basically, there is a need that’s a great stressor because if a mom has the stress she’s homeless, it’s hard for her to focus on a group if she has the other stressors.” [Facilitator]

A second challenge commonly identified by group facilitators was implementing materials with clients who were either illiterate, had literacy challenges, or learning disabilities. Some facilitators noted the need to accommodate for different reading levels by completing activity sheets out loud instead of having clients write out their responses. Facilitators suggested for future groups to provide more opportunities for clients to complete materials out loud to not single out clients who are uncomfortable with writing.

“So, with the manual we did have one participant in one cohort that did have difficulty with reading and writing...I didn't want that to become something that made her uncomfortable in the group and that she felt other people were aware of that...I just basically tried to read as much as I possibly could from each of the, you know, from the participant's manual, specifically, because of that reason. And I also, of course, the personal projects [QMS] I went over that.” [Facilitator]

One of the main challenges highlighted by facilitators was managing discussions during group. Facilitators aimed to create an environment where clients felt comfortable sharing, listening to their peers, and reviewing the information presented in group. Some facilitators felt this balance was harder to achieve their first time facilitating as they were becoming acquainted with the curriculum. Challenges that impacted finding a healthy equilibrium during group implementation included: side conversations between clients, family crises shared during groups, and clients who required additional time due to disabilities, learning, or English as a Second Language challenges.

"if someone is wanting to share something that is really personal or really intense or difficult, that if you were delivering this one-on-one, you could really spend some time focusing and really talking about that. But in a group setting, you might be able to spend some time talking about that to acknowledge and validate and to recognize just the importance of what someone shared, but you just can’t spend the whole time talking about that. And so, I think that inherently can feel really difficult as a facilitator to move on from that, and also being respectful to the other group members, and also knowing that we have material to cover.” [Facilitator]

## Discussion

The aim of this qualitative analysis was to determine the acceptability and appropriateness of the MB content, group format, and implementation for clients and facilitators. The results show that overall both clients and facilitators found the content and group format to be acceptable and appropriate. Clients not only spoke to how much they enjoyed the content and group setting, but also spoke to the effectiveness of the content and content delivery by providing examples of how they used core concepts outside of the group setting. Clients emphasized how well the content was delivered noting that facilitators explained concepts in a way that made them easy to understand. This could be due to the fact that facilitators are encouraged during training, supervision, and in the instructor manual to use their own examples and make adjustments as needed for their given audience.

Facilitators generally found the content to be universal to most populations, highlighting the appropriateness of MB for pregnant women of varying backgrounds. One exception was that facilitators found the material difficult to implement with women experiencing crises, housing instability, and/or trauma. Given the frequency that this was highlighted by facilitators, a logical next step may be to modify the MB instructor manual and training to include examples and suggestions that guide facilitators in working with women facing challenging life situations. Another area for improvement of the MB materials is to include more suggestions for facilitators delivering the intervention with participants who have learning and/or literacy challenges. This is currently covered during the MB training, but the frequency of facilitator comments on this topic suggests a need to add more information during both training and supervision.

Financial support was provided for transportation and child care to sites to minimize participant absences at group sessions. Participants voiced this support as being an important enabler of group attendance. This is consistent with findings from other studies examining group interventions delivered to women prenatally [19, 20]. Interestingly, child care was deemed necessary by participants regardless of whether they already had children of their own or not. Clients explained they often had child care responsibilities for other people’s children (including family members), and if child care was not provided they would have been unable to attend group. While transportation support was brought up as an enabler to attendance, transportation was also noted as a barrier to attendance for some clients. The support that was provided, while helpful, may not have been enough. These findings suggest that HV programs and other agencies implementing group-based perinatal interventions such as MB should allocate adequate resources for transportation and child care to increase attendance despite the cost associated with these supports.

Clients and facilitators noted it was sometimes difficult for clients to get out of the house or actively participate due to the discomforts and medical issues associated with pregnancy. Ensuring there is comfortable seating, several breaks, and flexibility in scheduling could assist in achieving higher attendance. Group size did not seem to impact participants’ overall enjoyment of sessions. The composition of groups, including the age of participants and if participants were first-time mothers or not, also did not seem to have a meaningful impact. Largely, participants were able to find common ground in pregnancy and shared lived experiences. The overwhelmingly positive feedback regarding the relationships built in groups exemplifies the importance of social support for pregnant women [19, 21–23]. This was also evidenced by the desire voiced by some participants for cohorts to go on longer.

Through the qualitative analysis, researchers looked at responses from clients and facilitators in the two study arms separately and looked for differences. Overall, there were not any notable differences in findings between the two study arms. Findings from the primary analysis of the study showed participants who received the intervention from paraprofessional HV staff had similar reductions in depressive symptoms as participants who received the intervention from MHPs [24]. These findings coupled with the findings from the qualitative analysis reported here make a strong case for the use of paraprofessionals to implement MB. This could lead to greater cost savings and accessibility to HV programs who may not have the staff or resources to employ MHPs, and is in line with a global push for using lay health workers to address disparities in mental health services [25].

### Limitations

There are a few limitations of this study. Interviews with clients were only conducted with individuals who attended at least one of the group sessions. While outside the scope of this study, in the future it could be beneficial to interview participants who did not attend group sessions to better understand barriers to attendance. Facilitator interviews were conducted after all cohorts were implemented at their site. This may have created challenges for facilitators in remembering each cohort they facilitated for those who implemented more than one. Lastly, one area that was not fully explored in this analysis was potential differences between facilitators who were employed through the agencies where MB was implemented and those who were not. This could have illuminated differences in implementation associated with facilitators’ relationship with the implementing agency.

## Conclusions

This study adds to the qualitative literature on client and facilitator perceptions of perinatal depression preventive interventions and the use of paraprofessionals delivering maternal and child health interventions. Given the mixed findings on the effectiveness of perinatal depression preventive interventions [7, 14], our findings can provide guidance to future intervention trials—including our own MB studies—on key considerations that may affect intervention implementation. Results from this qualitative study also indicate that pregnant women find paraprofessional-delivered interventions to be as acceptable and appropriate as interventions led by MHPs, thereby suggesting that future maternal and child health interventions could be tested using paraprofessionals.

## Supplementary information

**Additional file 1. **Quick Mood Scale. Description of data: Example page from the Mothers and Babies Participant Manual. Source: Degillio, A., Segovia, M., Leis, J., Tandon, S.D., Mendelson, T., Jensen, J., & Diebold, A. (n.d.). *The Mothers & Babies Program, A reality management approach; Participant manual.*

**Additional file 2. **Violet and Mary’s Days. Description of data: Example page from the Mothers and Babies Participant Manual. Source: Degillio, A., Segovia, M., Leis, J., Tandon, S.D., Mendelson, T., Jensen, J., & Diebold, A. (n.d.). *The Mothers & Babies Program, A reality management approach; Participant manual.*

**Additional file 3.** Mothers and Babies Group Program: Semi-Structured Interview for Intervention Participants. Description of data: Interviewee script for interviews with intervention clients.

**Additional file 4.** Mothers and Babies Group Program: Semi-Structured Interview for Group Facilitators. Description of data: Interviewee script for interviews with intervention facilitators.

**Additional file 5.** Codebook for Clients. Description of data: Codebook used for coding interviews with intervention clients.

**Additional file 6.** Codebook for Facilitators. Description of data: Codebook used for coding interviews with intervention facilitators.

## Data Availability

The datasets used and/or analyzed during the current study are available from the corresponding author on reasonable request.

## Abbreviations

CBT: Cognitive behavioral therapy
C-RCT: Cluster randomized controlled trial
HV: Home visiting
MB: Mothers and Babies Program
MHP: Mental health professional
PI: Principal Investigator
QMS: Quick Mood Scale
