# Supplementary material for: Acceptability and appropriateness of a perinatal depression preventive group intervention: a qualitative analysis
Source: BMC Health Serv Res. 2020 Mar 7;20:189. doi: 10.1186/s12913-020-5031-z (PMC7060621; doi:10.1186/s12913-020-5031-z)
Supplement: Supplementary file 1 — Additional file 1. Quick Mood Scale. Description of data: Example page from the Mothers and Babies Participant Manual. Source: Degillio, A., Segovia, M., Leis, J., Tandon, S.D., Mendelson, T., Jensen, J., & Diebold, A. (n.d.). The Mothers & Babies Program, A reality management approach; Participant manual. [file 12913_2020_5031_MOESM1_ESM.pdf]

# Quick Mood Scale

**Instructions:** Track your mood every day using the Quick Mood Scale. It will help you learn to be aware of how you feel, so that you can learn to have healthier moods and teach your baby to balance his/her moods.

- The seven columns represent each day of the week.
- Write down the date above each of the seven columns.
- Every night, before going to bed, circle the number (between 1-9), which indicates how you feel on that day. For example:
  - if your **mood is average** (neither high nor low) circle **number 5**
  - if it is **better than average**, circle **a number higher than 5**
  - if it is **worse than average**, circle **a number lower than 5**
- The number you choose will only reflect how you feel that day—there is no right or wrong answer. We find that it is easiest to keep the scale by the bed, so that before you go to bed, you can think about your day and rate your mood for the day.

| **Date:** | ______ | ______ | ______ | ______ | ______ | ______ | ______ |
|---|---|---|---|---|---|---|---|
| **Best Mood** | 9 | 9 | 9 | 9 | 9 | 9 | 9 |
| | 8 | 8 | 8 | 8 | 8 | 8 | 8 |
| | 7 | 7 | 7 | 7 | 7 | 7 | 7 |
| | 6 | 6 | 6 | 6 | 6 | 6 | 6 |
| **Average** | 5 | 5 | 5 | 5 | 5 | 5 | 5 |
| | 4 | 4 | 4 | 4 | 4 | 4 | 4 |
| | 3 | 3 | 3 | 3 | 3 | 3 | 3 |
| | 2 | 2 | 2 | 2 | 2 | 2 | 2 |
| **Worst Mood** | 1 | 1 | 1 | 1 | 1 | 1 | 1 |

**Optional Project:**

1. Discuss the Mothers and Babies Program with a friend or a family member. Talk about what you learned in today's session.
