# Supplementary material for: Acceptability and appropriateness of a perinatal depression preventive group intervention: a qualitative analysis
Source: BMC Health Serv Res. 2020 Mar 7;20:189. doi: 10.1186/s12913-020-5031-z (PMC7060621; doi:10.1186/s12913-020-5031-z)
Supplement: Supplementary file 2 — Additional file 2. Violet and Mary’s Days. Description of data: Example page from the Mothers and Babies Participant Manual. Source: Degillio, A., Segovia, M., Leis, J., Tandon, S.D., Mendelson, T., Jensen, J., & Diebold, A. (n.d.). The Mothers & Babies Program, A reality management approach; Participant manual. [file 12913_2020_5031_MOESM2_ESM.pdf]

Violet and Mary’s Days

Instructions: Both Violet and Mary have a 1 year old baby. Circle the number on each panel that represents what kind of mood you think each woman is having.

Violet’s DAY | **MARY’s DAY**

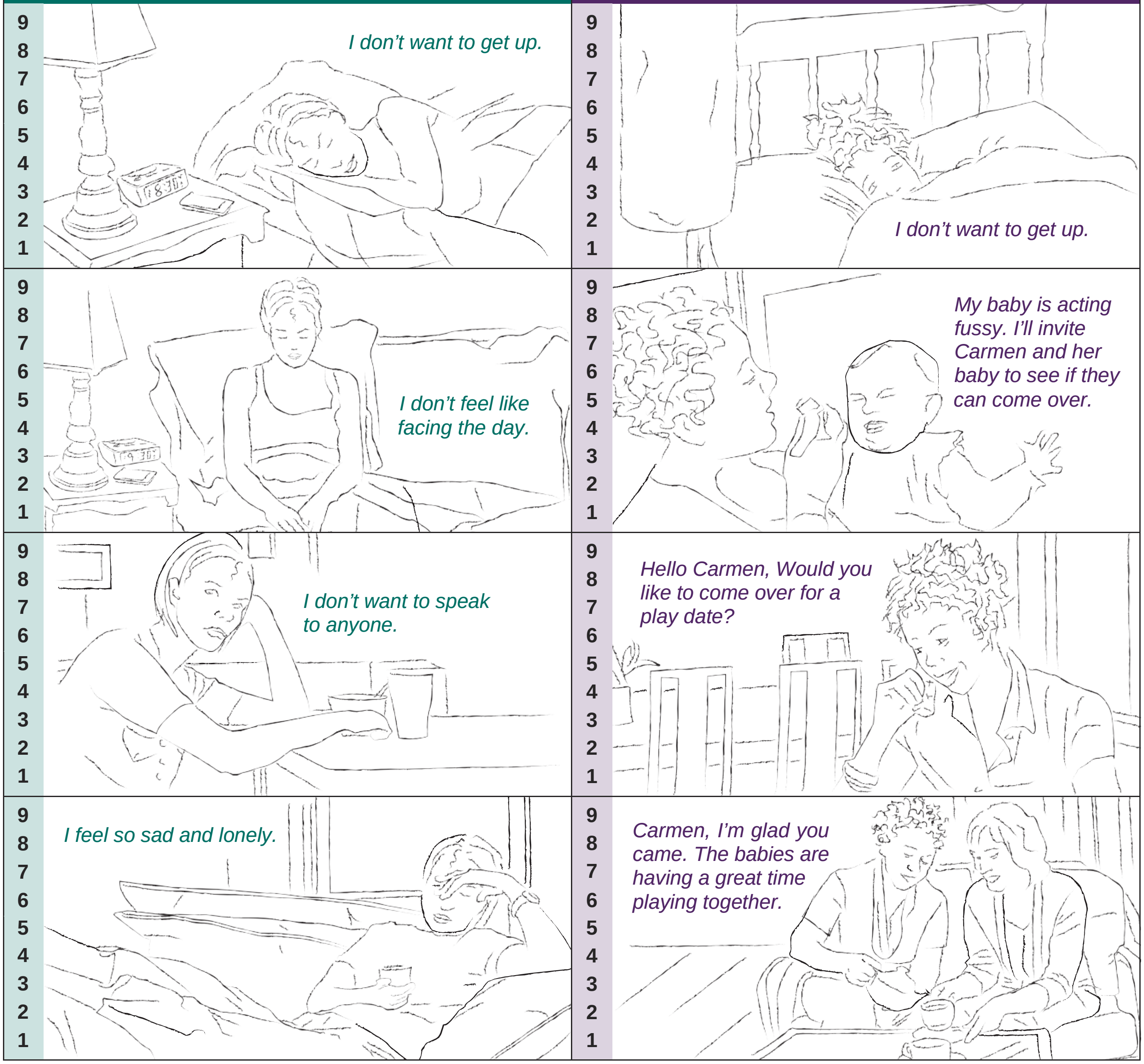

ILLUSTRATIONS BY JOSIE THOMPSON, THOMPSON DESIGN
