# Supplementary material for: Acceptability and appropriateness of a perinatal depression preventive group intervention: a qualitative analysis
Source: BMC Health Serv Res. 2020 Mar 7;20:189. doi: 10.1186/s12913-020-5031-z (PMC7060621; doi:10.1186/s12913-020-5031-z)
Supplement: Supplementary file 3 — Additional file 3. Mothers and Babies Group Program: Semi-Structured Interview for Intervention Participants. Description of data: Interviewee script for interviews with intervention clients. [file 12913_2020_5031_MOESM3_ESM.pdf]

Mothers and Babies Group Program: Semi-Structured Interview for Intervention Participants

I. *Informed Consent*

- Participants will have already signed a consent at the start of the research study which included the possibility of being randomly selected to be interviewed. Review signed consent form with participant.

II. *Interview Instructions*

- I am going to ask you some questions about your experiences participating the Mothers and Babies (MB) groups. I will begin with questions about your understanding of the Mothers and Babies information; next I will ask you a few questions about your satisfaction with your participation in the group; and finally I will ask about any challenges you faced attending the group session and if you have any suggestions for making the groups better. As we discussed in the consent form, I will audio-record our interview. At any point during the interview, you can ask me to turn off the recorder if you would like to make a comment(s) off the record. This interview will be transcribed and analyzed in order to help us improve the course. All the data will be de-identified, meaning we will use a study ID, not your name, and will be kept confidential.

III. *Interview Questions*

(a) Background

- When you first heard about the Mothers and Babies program, what led you to sign up for the group sessions?

(b) MB Information and Group Satisfaction

- Do you feel that the Mothers and Babies groups provided helpful information for you? Please explain.
- Can you highlight certain things that you think were most helpful?
- How well do you feel that you understood the material that the group leader was presenting to the group? (probe for reading level, trouble understanding the concepts, relevant to lifestyle, values, ad beliefs)
- How comfortable were you sharing information in the groups and/or participating in group activities? (Probe on group size and structure)
- Do you think the Mothers and Babies sessions helped you manage your mood and stress? Please explain.
- What was your favorite thing about participating in the Mothers and Babies groups?

(c) Challenges and Improvements

- What specific challenges did you experience while participating in the sessions? (probe for specific barriers: not enough time or too much time, group size, comfort in speaking with the group, completing personal projects)

- How many Mother and Babies group sessions did you attend out of the 6 total sessions? If you attended fewer than 6 sessions were there specific reasons you did not attend some sessions? (probe for specific barriers regarding traveling to the groups (e.g. Location of group, transportation to the group, and childcare support)

- Were there specific things that motivated you to come to groups? If yes, please explain.

- What do you think could be changed to make the groups better? Are there any topics you would have liked to talk about that were not addressed during the group sessions?

(d) General Feedback

- Is there anything else you would have liked to have been included in the groups? Is there any other feedback you would like to share?

Thank you so much for spending time to provide your feedback today!
