# Supplementary material for: Acceptability and appropriateness of a perinatal depression preventive group intervention: a qualitative analysis
Source: BMC Health Serv Res. 2020 Mar 7;20:189. doi: 10.1186/s12913-020-5031-z (PMC7060621; doi:10.1186/s12913-020-5031-z)
Supplement: Supplementary file 4 — Additional file 4. Mothers and Babies Group Program: Semi-Structured Interview for Group Facilitators. Description of data: Interviewee script for interviews with intervention facilitators. [file 12913_2020_5031_MOESM4_ESM.pdf]

Mothers and Babies Group Program: Semi-Structured Interview for Group Facilitators

I. *Interview Instructions*

- I am going to ask your some questions about your experiences implementing the Mothers and Babies (MB) Program in a group setting with participants of a home visiting program. I will begin with questions about the training and how well prepared you felt; next I will ask you a few questions about implementation; and finally I will ask about the participants' experience with the material. I will be audio-recording our interview. (If needed, remind the facilitator that they consented to this in the original consent form). At any point during the interview, you can ask me to turn off the recorder if you would like to make a comment(s) off the record. This interview will be transcribed and analyzed in order to help us improve the program. All the data will be de-identified, meaning we will use a study ID, not your name, and it will be kept confidential.

II. *Interview Questions*

(a) *Background*

- When you first heard about the Mothers and Babies Group Program, what led you to want to lead groups?

(b) *MB Training and Materials*

- Overall, how was your experience with the training? What did you find most helpful about the training? What was least helpful about the training? What changes, if any, would you recommend for the training?

- Did any of your perceptions about the Mothers and Babies Program change after the training? If so, how?

- There are two manuals that are used for the MB Program--an instructors manual and a participant manual. Do you have any feedback on these manuals in terms of things that worked well or not well with them?

(c) *MB Implementation*

- How many cohorts of the Mothers and Babies Program did you facilitate?

- What do you see as the main strengths of the Mothers and Babies Program?

- In general, were you able to implement the Mothers and Babies Program as planned? What specific challenges did you experience while setting up/delivering/implementing the sessions? (probe for specific barriers: scheduling groups, not enough time, people not showing up)

- What did you find to be the main strengths of delivering the Mothers and Babies Program information in a group setting? What were some of the challenges of delivering the material in a group setting?

(d) *MB Supervision*

- How was your experience with supervision? Did you feel you received the right amount of supervision?

- Please comment on the strengths and the limitations of the supervision you received for implementing the Mothers and Babies sessions.

- Were there things that came up during the Mothers and Babies sessions that you felt unprepared to handle? (Probe for past trauma, current abuse?)

(e) MB Acceptability and Effectiveness

- Do you feel that the Mothers and Babies Program was accessible and relevant to the participants? How well do you feel that the participants understood the material? (probe for reading level, trouble understanding the concepts, whether they did their personal projects between sessions)

- We planned for each session to last approximately two hours. In general, were you able to stick to the guidelines? (Probe: which parts took longer, which parts took less time)

- How relevant was the content to the mission of the home visiting program?

- Do you think the Mothers and Babies groups helped the participants manage their mood and stress?

- Were there stressors or risk factors that the participants were experiencing that the Mothers and Babies group sessions didn't address sufficiently? What were these?

[ASK THE FOLLOWING QUESTIONS IN SECTION F ONLY FOR HOME VISITING STAFF]

(f) Self-efficacy

- Do you feel more confident discussing depression with the women you work with as a result of facilitating the Mothers and Babies Groups? Do you feel more confident discussing mental health in general?

- Have you found that you're able to recognize symptoms of depression with your home visiting participants as a result of your experience with the Mothers and Babies Program?

- Have you noticed any other changes in your work with families as a result of the Mothers and Babies Program?

(g) General Feedback

- Overall, how would you rate the Mothers and Babies Group Program on a 1-5 scale with 5 being Excellent and 1 being Poor? (When answering, please think about ease of implementation and course content)

- Tell me how you think we can improve the delivery of the Mothers and Babies Program in a group setting.

Thank you so much for spending time to provide your feedback today!
