# Supplementary material for: Acceptability and appropriateness of a perinatal depression preventive group intervention: a qualitative analysis
Source: BMC Health Serv Res. 2020 Mar 7;20:189. doi: 10.1186/s12913-020-5031-z (PMC7060621; doi:10.1186/s12913-020-5031-z)
Supplement: Supplementary file 5 — Additional file 5. Codebook for Clients. Description of data: Codebook used for coding interviews with intervention clients. [file 12913_2020_5031_MOESM5_ESM.pdf]

<u>Codebook for Clients</u>

1.0 Background knowledge/motivation for joining groups
2.0 Group satisfaction
- 2.1 Satisfaction with information presented in group
- 2.2 Dissatisfaction with information presented in group
- 2.3 Understanding of material
- 2.4 Lack of understanding of material
- 2.5 Comfort of being in group
- 2.6 Discomfort of being in group
- 2.7 Effectiveness of MB
- 2.8 Ineffectiveness of MB
- 2.9 Benefits of group

3.0 Attending groups
- 3.1 Challenges during groups
- 3.2 Challenges completing person projects
- 3.3 Number of sessions attended
- 3.4 Barriers to going to groups
  - 3.4.1 Barriers related to transportation
  - 3.4.2 Barriers related to childcare
  - 3.4.3 Barriers related to scheduling
- 3.5 Motivations for going to groups
  - 3.5.1 Desire to learn
  - 3.5.2 Social support
  - 3.5.3 Food
- 3.6 Suggestions for improvements
- 3.7 Satisfaction with group size
- 3.8 Dissatisfaction with group size
- 3.9 Feedback related to group structure
- 3.10 Length of cohorts
- 3.11 Length of sessions

4.0 General feedback
