# Supplementary material for: Acceptability and appropriateness of a perinatal depression preventive group intervention: a qualitative analysis
Source: BMC Health Serv Res. 2020 Mar 7;20:189. doi: 10.1186/s12913-020-5031-z (PMC7060621; doi:10.1186/s12913-020-5031-z)
Supplement: Supplementary file 6 — Additional file 6. Codebook for Facilitators. Description of data: Codebook used for coding interviews with intervention facilitators. [file 12913_2020_5031_MOESM6_ESM.pdf]

Codebook for Facilitators

1.0 Background knowledge/interest in facilitating MB groups
2.0 Training and material
    2.1 Positives about the training
    2.2 Critiques of the training
    2.3 Perceptions of MB that changed after training
    2.4 Positive feedback related to Instructor Manual
    2.5 Negative feedback related to Instructor Manual
        2.5.1 Feedback related to translated Instructor Manual
    2.6 Positive feedback related to Participant Manual
    2.7 Negative feedback related to Participant Manual
        2.7.1 Feedback related to translated Participant Manual
3.0 MB Implementation
    3.1 Number of cohorts facilitated
    3.2 Strengths/successes with implementation
        3.2.1 Social support, relationships
    3.3 Challenges/barriers to implementation
        3.3.1 Challenges related to intervention implementation
            3.3.1.1 Challenges related to recruitment
            3.3.1.2 Challenges related to scheduling
            3.3.1.3 Challenges related to group structure
        3.3.2 Challenges related to research requirements
            3.3.2.1 Challenges related to facilitator and staff time
4.0 Supervision
    4.1 Strengths of supervision
    4.2 Limitations of supervision
    4.3 Ability to respond to issues brought up in group
    4.4 Issues with scheduling supervision
5.0 MB Acceptability and Effectiveness
    5.1 Acceptability for participants
    5.2 Unacceptability for participants
    5.3 Acceptability for HV program
    5.4 Unacceptability for HV program
    5.5 Length of sessions
    5.6 Effectiveness of MB
    5.7 Ineffectiveness of MB
6.0 Self efficacy
    6.1 Confidence in discussing depression/mental health
    6.2 Ability to recognize symptoms of depression
    6.3 Changes in work with families as result of MB
7.0 General
    7.1 Rating of MB groups
    7.2 Areas for improvement
